# Cyclin D1 Expression and the Inhibitory Effect of Celecoxib on Ovarian Tumor Growth *in Vivo*

**DOI:** 10.3390/ijms11103999

**Published:** 2010-10-19

**Authors:** Wei Li, Hong-Ru Jiang, Xiao-Li Xu, Jie Wang, Jun Zhang, Mei-Lin Liu, Ling-Yun Zhai

**Affiliations:** Department of Gynecology, Nanjing Medical University of Hangzhou Hospital, 261 Huansha Road, Hangzhou, Zhejiang, 310006, China; E-Mails: hongrujiang@tom.com (H.-R.J.); joyce.xiaolixu@gmail.com (X.-L.X.); hongsheqiao@sina.com (J.W.); viola0116@163.com (J.Z.); lml13588722926@163.com (M.-L.L.); 494610422@qq.com (L.-Y.Z.)

**Keywords:** Celecoxib, cyclin D1, ovarian cancer, proliferation, apoptosis

## Abstract

The report aims to investigate the relationship between the expression of cyclin D1 and Cyclooxgenase-2 (COX-2), thus to explore the molecular mechanisms of the antitumor efficacy of Celecoxib, a COX-2 inhibitor. Human ovarian SKOV-3 carcinoma cell xenograft-bearing mice were treated with Celecoxib by infusing gaster (i.g.) twice/day for 21 days. The mRNA levels of COX-2 and cyclin D1 were determined by RT-PCR. The expression of cyclin D1 at the protein level was detected by immunohistochemistry, while COX-2 protein expression was determined by Western blot. A high-dose of Celecoxib (100 mg/kg) significantly inhibited tumor growth (*P* < 0.05), and the expression of cyclin D1 was reduced by 61%. Celecoxib decreased the proliferation cell index by 40% (*P* < 0.001) and increased apoptotic index by 52% (*P* < 0.05) in high-dose Celecoxib treated group. Our results suggest that the antitumor efficacy of Celecoxib against ovarian cancer in mice may in part be mediated through suppression of cyclin D1, which may contribute to its ability to suppress proliferation.

## 1. Introduction

Ovarian cancer is an insidious disease that has few specific symptoms in the early stages and most women with this disease present with an advanced stage at the time of diagnosis. The current management of advanced epithelial ovarian cancer generally includes cytoreductive surgery followed by combination chemotherapy, but the long-term survival of ovarian cancer patients remains unsatisfactory [1]. Therefore, searching for new alternative agents for the prevention and treatment of ovarian cancer is essential.

Cyclooxgenase-2 (COX-2) is inducible by inflammatory stimuli, including cytokines, growth factors, and tumor promoters, and is upregulated in a variety of malignancies and favors the growth of malignant cells by stimulating proliferation and angiogenesis [2,3]. Recently, large number of studies demonstrated that COX-2 is over-expressed in ovarian cancer [4–6]. Furthermore, Arico *et al*. found that COX-2 can induce angiogenesis via vascular endothelial growth factor (VEGF) and prostaglandin production and can also inhibit apoptosis by inducing the antiapoptotic factor Bcl-2 as well as activating antiapoptotic signaling through Akt/PKB (one of the serine/threonine kinases) [7]. These results suggest that COX-2 plays an important role in the generation and progression of solid tumors, and inhibition of COX-2 may inhibit the growth of a variety of solid malignancies. In addition, many reports have revealed that COX-2 inhibitor therapy could be beneficial in the prevention and/or treatment of ovarian cancer [8–10]. Celecoxib, a COX-2 inhibitor, inhibits meningioma growth *in vivo* at plasma levels achievable in humans, and the tumors treated with Celecoxib had low expression of COX-2 and were less vascular with increased apoptosis [11]. The research indicated that Celecoxib has a direct effect on COX-2 protein expression in an *in vivo* model of spontaneous metastatic breast cancer [12], and the COX-2 expression decreased correspondingly with an increasing dose of Celecoxib in human colorectal adenocarcinoma cells [13]. One of the main anticancer mechanisms of Celecoxib is to decrease production of prostaglandins in the COX-2 expressing neovasculature of tumors, resulting in an inhibition of proliferation and induction of apoptosis in both the vasculature and the tumor [14].

Cyclin D1, one of the cell cycle proteins, affects the proliferation of a cell. The expression of cyclin D1 might be responsible for progression and/or ultimately tumorigenesis of human ovarian cancer epithelial tissues [15]. In epithelial ovarian cancer, overexpression of cyclin D1 has been associated with decreased survival in patients [16]. Therefore, it has been hypothesized that a decrease of cyclin D1 could be potentially effective in inhibiting proliferation of tumor cells. In this study, to discuss the relationship between COX-2 and cyclin D1, we investigated the potential effect of Celecoxib on the growth of malignant ovarian tumors in a SKOV-3 cells mouse xenograft model and aimed to elucidate the molecular mechanism of its antitumor effect in relation to cyclin D1.

## 2. Results and Discussion

### 2.1. Inhibition of Ovarian Cancer Growth

To test whether Celecoxib could inhibit ovarian cancer growth, we used the human ovarian carcinoma cell line SKOV-3. SKOV-3 cells were implanted into the subcutaneous growth, so that changes in tumor growth could be easily monitored. The tumor growth increased throughout the period examined in the vehicle-treated group. After three weeks of treatment with high dose of Celecoxib and 5-Fu, mean tumor volumes in the group treated with Celecoxib was 2425 ± 71 mm^3^, in mice with 5-Fu was 2230 ± 12 mm^3^. Under similar conditions, the mean tumor volume in control mice was 2900 ± 55 mm^3^. Tumor growth was significantly reduced during the end of treatment period with high doses of Celecoxib and 5-Fu treatment (*P* < 0.05, all). No toxicity was observed in any of the animals as measured by weight gain/loss as well as gross pathological examination of the gastrointestinal tract of the animals at necropsy.

### 2.2. Celecoxib Decreases COX-2 and Cyclin D1 Expression in Tumors

**Figure 1:**
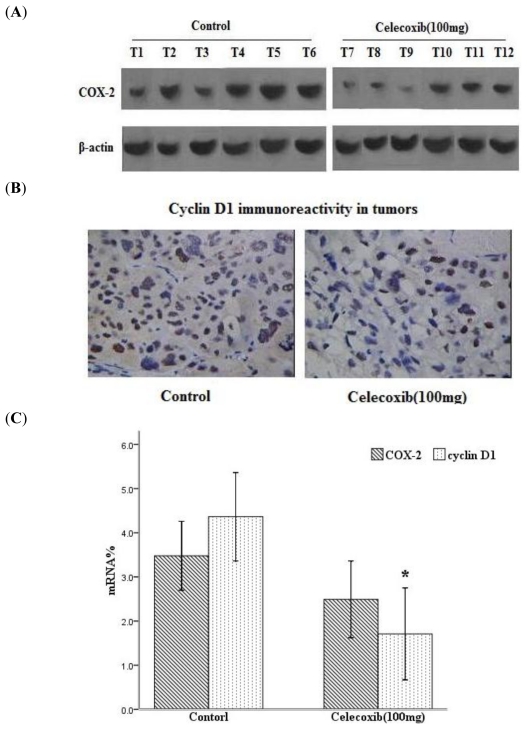
Effects of Celecoxib on COX-2 and cyclin D1 activation in SKOV-3 xenograft tumors: (**A**) Western blot analysis of SKOV-3 tumors treated with a high-dose of Celecoxib (100 mg/kg). Tumor cells lysate protein from six control mice (T1–T6) and six Celecoxib-treatment mice (T7–T12); (**B**) Representative pictures of cyclin D1 immunohistochemical staining of tumors. Magnification is ×400; (**C**) mRNA levels of COX-2 and cyclin D1 detected by RT-PCR. The results show that both COX-2 and cyclin D1 expression in tumors were decreased in the high-dose Celecoxib treatment group. *P < 0.05 compared with control, error bars indicate SE.

COX-2 expression was evaluated by Western blot and RT-PCR. These analyses revealed that after Celecoxib treatment (100 mg/kg), the expression of the COX-2 protein was substantially decreased in mouse ovarian carcinoma (Figure 1A). To evaluate the expression of cyclin D1 in Celecoxib-treated mice, protein and mRNA changes in drug-treated xenograft tumors were detected by immunohistochemistry and RT-PCR analysis. Immunohistochemistry analysis revealed that quantification of cyclin D1-postive cells showed 47.00% of cells in the control *versus* 28.33% of cells (*P* < 0.05) in the group treated with a high-dose of Celecoxib (Figure 1B). As shown in Figure 1C, Celecoxib (100 mg/kg) significantly decreased the mRNA expression levels of cyclin D1 compared with the control (*P* < 0.05), and it also decreased the expression of COX-2 mRNA levels.

### 2.3. Effect on VEGF Production

**Figure 2:**
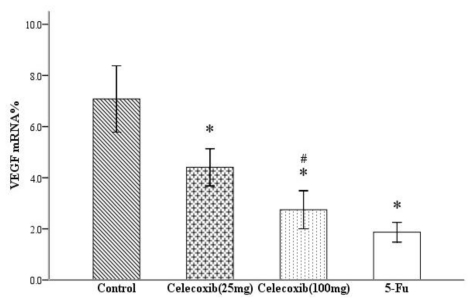
Effects of the drugs on the expression of VEGF. mRNA levels of VEGF were decreased in treatment groups. *P < 0.05 compared with control, ^#^*P* < 0.05 compared with Celecoxib-25 mg group, error bars indicate SE.

In this report, we measured VEGF levels in xenograft tumors by real-time PCR analysis. Four molecular isoforms of VEGF are generated by alternative splicing, rendering proteins containing 206-, 189-, 165- and 121-amino acid residues [17]. Although VEGF 206 transcripts were not amplified, VEGF 189, 165 and 121 were routinely detected in this series of ovarian cancer. Real-time PCR analysis indicated the Δ*CT* (cycle threshold, = *CT*_selected gene_ − *CT*_β-actin_) of VEGF in the four groups shown in Table 1. Comparing the results of the control and treatment groups, the expression levels of VEGF mRNA were significantly suppressed in all treatment groups (all *P* < 0.05, Figure 2). As the dose of Celecoxib increased, the inhibition of mRNA expression was more pronounced (*P* < 0.05). The results showed a dose-dependent inhibition of the expression of VEGF mRNA after treated with Celecoxib.

### 2.4. Celecoxib Inhibits Tumor Cell Proliferation and Induces Apoptosis

**Figure 3:**
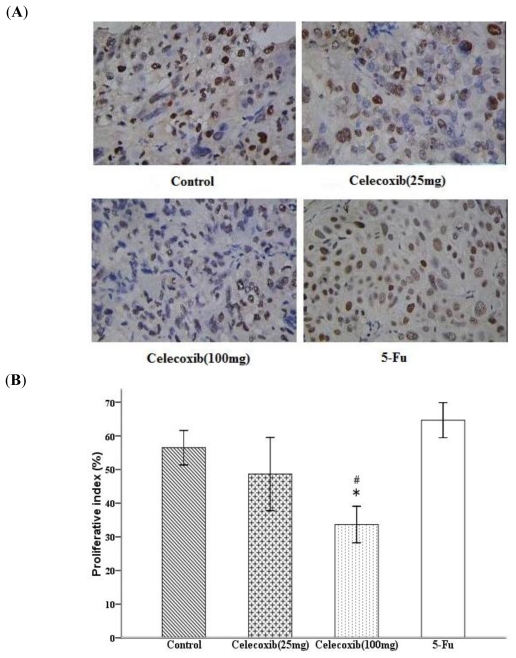
COX-2 inhibitor effect on the proliferation of tumors: (**A**) representative photomicrograph of Ki-67 staining of tumor tissue; (**B**) proliferation index. Quantitative data for the proliferation index is shown as percent of Ki-67-postive cells. Proliferation index illustrates the proliferation inhibition of Celecoxib on tumors. *P < 0.05 compared with control, ^#^*P* < 0.05 compared with low-dose Celecoxib treated group, error bars indicate SE.

To further characterize the antitumor activity of COX-2 inhibition, we compared tumor tissue proliferation index and apoptosis index of Celecoxib/5-Fu-treated mice with vehicle-treated control mice. The quantification of the Ki-67-positive cells in the tumors showed that high-dose Celecoxib treatment to nude mice results in a decrease in proliferation index compared with control (Figure 3A). As shown in Figure 3B, statistically significant differences in tumor proliferation inhibition were noted between the high-dose Celecoxib treatment and control group (33.67 ± 2.11% *versus* 56.50 ± 2.00%, *P* < 0.05). In addition, a statistically significant difference was observed between the low-and high-dose Celecoxib treatment groups (48.67 ± 4.21% *versus* 33.67 ± 2.11, *P* < 0.05). The results of this experiment comparing the proliferation index of tumors treatment with low-and high-dose Celecoxib showed a trend toward a dose-dependent proliferation inhibition response.

**Figure 4:**
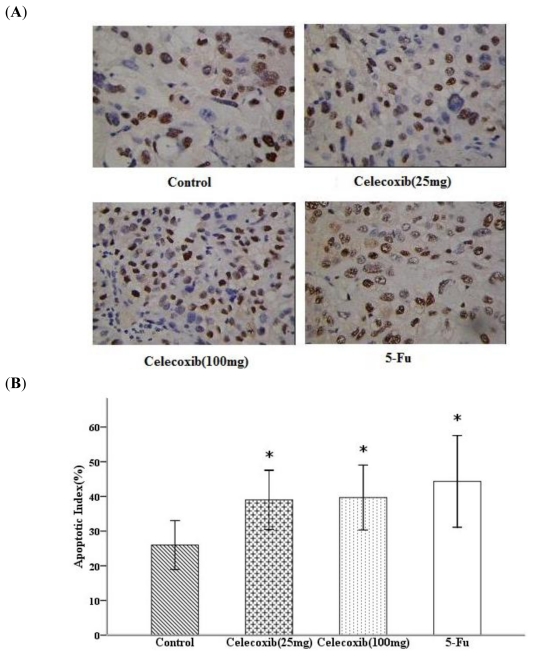
COX-2 inhibitor effect on the apoptosis of tumors. (**A**) Apoptotic cells of tumor tissue. Light microscopic image of TUNEL-positive cells visualizing apoptosis from ovarian tumor sections isolated from vehicle, 5-Fu and 25, 100 mg/kg Celecoxib-treated mice. Brown, apoptotic cells. All images are representative of five standardized fields from six separate mice. Magnification ×400. (**B**) Apoptotic index. Quantitative data for apoptotic index is shown as percent TUNEL-postive cells. Apoptotic index illustrates the apoptotic indution of Celecoxib on tumors. *P < 0.05 compared with control, error bars indicate SE.

To evaluate the extent of apoptosis in tumor tissue in a cancer-bearing mouse model, apoptotic cells were stained by the TUNEL method and the number of apoptotic-positive cells was counted in a high-power field. We observed an increase in apoptotic-positive cells in Celecoxib/5-Fu-treated tumor sections as compared with control tumor sections (Figure 4A). The index of apoptotic cells was significantly higher in the Celecoxib and 5-Fu treatment groups than in the control group (all *P* < 0.05, Figure 4B).

### 2.5. Discussion

The central finding in the present study was that Celecoxib suppressed the growth of human ovarian SKOV-3 carcinoma xenografts in athymic nude mice without any noticeable toxicity. High-dose Celecoxib showed greater antitumor efficacy than low-dose Celecoxib, and the antitumor effect of Celecoxib was associated with a decrease in proliferation index as observed by reduced cyclin D1 expression, a increase in apoptosis, and a strong inhibition of tumor angiogenesis as observed by VEGF analyses.

There is ample evidence to suggest an important role for COX-2 in cancer. Many reports indicate that COX-2 is up-regulated [4–6] and its expression is an independent prognostic factor [18] in human ovarian carcinoma. These results suggest that COX-2 plays an important role in the generation and progression of ovarian cancer, and inhibition of COX-2 may restrain the growth of ovarian cancer. Treatment with Aspirin caused cell growth inhibition and induces apoptosis in ovarian cancer cell lines, and also caused a time-dependent regression of xenograft tumors in mice [8]. In ovarian carcinoma cells, treatment with the COX-2 specific inhibitor NS-398 reduced Lipse Activator (LPA)-induced promatrix metalloproteinase-2 (proMMP-2) protein expression and activation and blocked MMP-dependent motility and invasive activity [19]. Other reports also revealed that selective COX-2 inhibition suppressed the growth of OVCAR-3 xenograft tumors of ovarian cancer [8,9]. The views that COX-2 is up-regulated in ovarian carcinoma and COX-2 inhibitor can restrain the growth of human ovarian cancer xenograft tumors are widely accepted by researchers. However, the molecular mechanism of their antitumor effect is still in discussion.

Cyclin D1, which correlates with enhanced proliferation, is one of the cell cycle proteins responsible for transition to the S phase (DNA synthesis) of the cell cycle, and its overexpression is associated with malignant transformation. Research has demonstrated that an estimated 26% of sporadic epithelial ovarian cancers overexpress cyclin D1 [20]. Another study also showed that cyclin D1 plays a dominant role in regulating cell cycle progression in ovarian cancer cells and that degradation of cyclin D1 is sufficient to induce G1 cell cycle arrest [21]. In this study we investigated the expression of cyclin D1 in ovarian carcinoma xenografts and the relationship between the antitumor effect of Celecoxib and the expression of cyclin D1. We investigated this relationship because research had previously reported that COX-2 [4–6] and cyclin D1 [20] were both up-regulated in ovarian cancer. In ovarian cancer SKOV3 cells, the expression of COX-2 protein was downregulated by Celecoxib treatment [22]. Cyclin D1 is rarely mutated, but its overexpression confers a selective growth advantage and hence acts as driver of neoplastic growth in various cancers [23]. Therefore, we suspected that there was a connection between COX-2 and cyclin D1. A corresponding decrease in cyclin D1-positive cells as well as their mRNA and protein expression by Celecoxib (100 mg/kg) was also observed. The results of our study manifested that there is a parallel decrease in the mRNA expression level of COX-2 and cyclin D1 in response to high-dose Celecoxib treatments, which confirmed our suppose. The discovery raised the possibility that Celecoxib inhibits the expression of Cyclin D1 by a COX-2 dependent mechanism and that up-regulation of cyclin D1 in ovarian cancer is induced by COX-2. In the case of celecoxib, the modulation of cyclin D1 levels appears to be a unique response because it was only observed in some cancer cell lines and it seems to be COX-2 dependent because it occurs in COX-2 overexpressing cell lines. Schiffmann *et al.* demonstrated that treatment of COX-2-negative HCT-116 human colon cancer cells with celecoxib reduced cyclin D1 expression, while in COX-2-overexpressing HCA-7 cells, celecoxib and methylcelecoxib (lack COX-2-inhibitory activity) caused a weaker degradation of cyclin D1 expression [24]. In addition, Celecoxib inhibited growth of SW480 human colon cancer cells through inhibiting subcellular cGMP-phosphodiesterase (PDE) enzymatic activity associated with a decrease in cellular levels of cyclin D1, while a high concentration of the COX-2 specific inhibitor rofecoxib did not inhibit growth of SW480 cells [25]. This research in colon cancer cells manifested that COX-independent mechanisms may play a distinct role in the anti-proliferative effect of Celecoxib.

Unchecked cell proliferation is necessary for solid tumor growth and progression. Cancer cells often have a selective growth advantage due to deregulation of cell cycle proteins, causing aberrant growth signaling that drives tumor development [26,27]. In this study, we have shown that the tumor growth inhibition by Celecoxib was accompanied by a decrease in proliferation index, and that high-dose Celecoxib exhibited greater efficacy in proliferation inhibition of human ovarian carcinoma SKOV-3 cells. The results suggested that Celecoxib may inhibit cell cycle progression through the G1-S transition in SKOV-3 cells *in vivo* by decreasing the expression of cyclin D1 as one of its potential antiproliferative mechanisms. Celecoxib-caused inhibition of tumor cell proliferation could be associated with G1 phase arrest during cell cycle progression. The hypothesis to explain the relationship between the decrease of cyclin D1 and the G1 cell cycle arrest is that the loss of cyclin D1 results in recruitment of p21 to cyclin E2-cdk2 complexes, inhibiting cdk2 activity, which prevents pRb hyperphosphorylation, and that the E2F promoters remain repressed by the bound pRb complex, resulting in G1 arrest [21]. The antiproliferative effects of Celecoxib could be attributed to the inhibition of cell survival signaling in ovarian cancer. Similar results could not be observed after treatment with Celecoxib in rat prostate cancer cells [28].

In addition to inhibiting proliferation, either low-dose or high-dose Celecoxib induced apoptosis in tumor cells compared with vehicle-treated group. Apoptosis is a multistep process and an increasing number of genes have been identified to be involved in the control or execution of apoptosis [29]. Recently, Uddin *et al.* [10] demonstrated that COX-2 inhibition induced apoptosis via inactivation of pAKT, resulting in disruption of mitochondrial membrane potential, which in turn leads to release of cytochrome C into the cytosol. Release of cytochrome C was associated with activation of caspase activity, eventually resulting in apoptosis. Celecoxib effectively inhibited cell growth and induced apoptosis in human ovarian cancer cells dependent upon the functional status of p53 [30]. Thus, a clear picture of the exact pathways by which COX-2 inhibitors induce apoptosis has yet to emerge.

Ovarian cancer growth is angiogenesis-dependent [31,32], and secretion of proangiogenic growth factors such as VEGF is of prognostic value [33,34]. Strong VEGF expression was suggested to play an important role in the tumor progression of ovarian carcinoma [35]. VEGF is a contributing factor in angiogenesis, a line of evidence reveals that the elevated COX-2 expression correlates with increased VEGF level in human ovarian cancer [36,37]. COX-2 can induce angiogenesis via VEGF [7]. In this study, Celecoxib strongly decreased the level of VEGF mRNA expression in ovarian SKOV-3 carcinoma xenografts both in the high-and low-dose drug treatment group and showed a dose-dependent inhibition of the expression of VEGF mRNA. The decrease in tumor-associated VEGF by Celecoxib could be one of the important mechanisms in controlling angiogenesis and causing an inhibition of overall tumor growth. Together, our findings suggested that the antiangiogenic potential of Celecoxib could be explored for lowering the risk of and preventing the growth and progression of ovarian cancer.

## 3. Experimental Section

### 3.1. Human Ovarian Tumors in Nude Mice

SKOV-3 cells were used for tumor growth studies *in vivo*. The SKOV-3 cells were purchased from China Type Culture Collection and grown in the recommended media under standard condition. SKOV-3 cells were inoculated subcutaneously into the right axillary region (5 × 10^6^ cells) of female BALB/cA nude mice (nu/nu, 6–7 weeks old). When the average tumor reached around 141–147 mm^3^, the mice were randomly separated into four groups (6 mice in each group). Mice were treated every other day by oral gavage with Celecoxib (25 mg/kg and 100 mg/kg i.g. twice/day) or vehicle (i.g. twice/day), i.p. injection with 5-Fu (20 mg/kg i.p. once/day), respectively from d1 to d21. All methods used in this study were approved by the Animal Subjects Programs of Nanjing Medical University and conform to Health guidelines and public law of the People’s Republic of China. Celecoxib was purchased from Pfizer Pharmaceutical Co. (Dalian, China), and 5-Fu was purchased from Xudong Haipu Pharmaceutical Co. (Shanghai, China). Celecoxib was suspended in vehicle (5% methylcellulose and 0.025% Tween 20) at appropriate concentrations and orally administered to the mice. The tumor dimensions were measured twice a week using a linear caliper, and tumor volume was calculated using the equation *V* (mm^3^) = *a* × *b*^2^/2, where *a* is the largest diameter and *b* is the smallest diameter. Tumor growth was evaluated by the inhibition rate as assessed by the formula: *IR* = *C* − *T*/*C* × 100%, where *IR* is the mean inhibition rate, *T* is the mean tumor volume in the treatment group and *C* is the mean tumor volume in the control group. The animals were weighed weekly throughout the experiment. On day 28, all of the mice were sacrificed, and tumor tissue samples were collected and then fixed in 10% phosphate-buffered formalin solution for immunohistology or snap-frozen in liquid nitrogen before storage at −80 °C until analysis.

### 3.2. Real-Time PCR

Total RNA was extracted using TRIzol reagents (Life Technologies), according to the manufacturer’s instructions. Isolated RNA was electrophoresed through 1.0% agarose-formaldehyde gels to verify the quality of the RNA. The first strand cDNA was generated by reverse transcription. After a sufficient amount of cDNA was obtained, we performed PCR amplification using a real-time PCR cycler (7500 ABI, USA). COX-2, cyclin D1 and VEGF 189/165/121 were routinely detected in this series of ovarian cancer. The sequences of PCR primers were: COX-2, 5′-AGACAGCGACGCCAAGCCAC-3′ and 5′-GGCGAGCGCGCAAAACCAAA-3′; VEGF (121), 5′-ACTCGGATGCCGACACGGGA-3′ and 5′-CCTGGCCTTGCTTGCTCCCC-3′; VEGF (165), 5′-CCAGGATCCTCTGCCCGCCT-3′ and 5′-GCGGCTTCCGGCACCTACAG-3′; VEGF (189), 5′-GGCAAAAGTTGCGAGCCGCC-3′ and 5′-TGGATGGACCGGGAGCAGGG-3′; Cyclin D1, 5′-AAGCCGCTTTTCCACGGCGA-3′ and 5′-AGGCTCCCCGGCTTCCACTT-3′; β-actin, 5′-GGGTGACGAGGCCCAGAGCA-3′ and 5′-GGGGCCACACGCAGCTCATT-3′.

The amplification system included 50 μL of SYBRGreen Mix (32.5 μL), ddH2O (14.5 μL), cDNA (2 μL), forward primer (0.5 μL) and reverse primer (0.5 μL). The reaction conditions were as follows: Stage 1, 50 °C for 2.00 min (1 cycle); Stage 2, 95 °C for 5.00 min (1 cycle); Stage 3, 95 °C for 0.25 min followed by 60 °C for 0.75 min (40 cycles); Stage 4, 95 °C for 0.25 min firstly, then 60 °C for 1.00 min, and lastly, 95 °C for 0.25 min followed by 60 °C for 0.25 min (1 cycle).

The results of real-time PCR were analyzed by the DCT method: Δ*CT* = *CT*_selected gene_ − *CT*_β-actin_, *RQ* (Relative Quantitation) = 2^−Δ^*^CT^* × 100%. The results of real-time PCR were presented as the ratio between the selected genes and β-actin transcripts.

### 3.3. Western Blot

Lysates (40 μg of protein/lane) were analyzed by SDS-PAGE on 12% Tris-glycine gels. Protein was electrotransferred to nitrocellulose membranes and blocked with a solution of PBS containing 5% milk and 0.1% Tween 20. Bands were detected using chemiluminescent detection reagents (GE healthcare, code: RPN2106). Blots were probed with a goat polyclonal antibody against COX-2 (Beijing biosynthesis biotechnology Co., China. code: BS-0732R) followed by a peroxidase-conjugated antigoat (abcam), respectively. After incubation, antibodies were washed in PBS and 0.1% Tween 20. Bands were detected using chemiluminescent detection reagents (GE healthcare, code: RPN2106).

### 3.4. TUNEL

Apoptosis was measured in tissue sections by the terminal deoxyribonucleotidyl transferase-mediated dUTP-biotin nick end labeling (TUNEL) assay as described by Gavrieli *et al.* [38] with some modifications. In this study, apoptosis was detected by the TUNEL kit (Chemicon Co. China). The tissue samples were fixed in 4% paraformaldehyde (PFA) for 24 h, dehydrated, and embedded in paraffin in the conventional manner. The paraffin-embedded tissues were cut into 4 μm-thick sections. After deparaffinization in a graded alcohol series, the tissue sections were covered with 20 μg proteinase K/mL PBS (−) for 15 min at room temperature, followed by blocking of endogenous peroxidase activity. The samples were then incubated with TdT enzyme and biotin-16-dUTP in TdT buffer containing 0.01% bovine serum albumin for 1.5 h at 37 °C in a humidity chamber. Biotin-16-dUTP nucleotides that had been incorporated into DNA fragments were detected using the ABC method with DAB as the chromogen. In each tissue specimen, five high-power fields (×400 magnification) were randomly selected, the apoptotic index (*AI*) was calculated in these fields as the percentage of positive cells, given by the following equation [39]:

AI=(number of positive cells/total number of cells)×100%

### 3.5. Immunohistochemistry

Proliferation index was evaluated by staining for Ki-67. At the same time, cyclin D1 protein was also detected by immunohistochemistry. Tumors were fixed in 10% neutral buffered formalin for 24–48 h prior to being embedded in paraffin. After deparaffinization, the tissue sections were heated at 121 °C for 15 min in 10 mM TrisHCl with 1 mM EDTA (pH 9.0). Endogenous peroxidase was blocked with 3% hydrogen peroxide in methanol for 10 min at room temperature. The samples were incubated with Ki-67 antibody (clone MIB-5 (M7248)) or cyclin D1 antibody (Santa Cruz Biotechnology, USA) for 90 min at room temperature. Then, the sections were incubated in EnVision reagent for 40 min and DAB/H2O2 for 8–12 min at room temperature. Proliferation was assessed by counting the number of Ki-67 positively staining nuclei and total number of cancer cells at ×400 magnification in five representative regions of the tumor. Results are expressed as the proportion of positively staining cells over the total number of cells. For evaluation of the cyclin D1 staining, the tissues were scored for the protein by assessing the site of positive staining in the nucleus or cytoplasm. The status of nuclear expression of cyclin D1 was assessed by determining the percentage of positive cells stained in five fields of each tissue section at ×400 magnification.

### 3.6. Statistical Analyses

Statistical analysis was performed with SPSS software (SPSS Standard version 17.0, SPSS). Statistical significance between control and treated groups was determined by Student’s *t*-test. We used a Dunnett’s test for evaluation of the inhibitory activity on tumor growth. All the experimental data were expressed as means values ± SE. Results were considered statistically significant when *P* values < 0.05.

## 4. Conclusions

The present findings provide evidence for antiproliferative, apoptosis-inducing and antiangiogenic effects of Celecoxib, which were associated with the inhibition of human ovarian SKOV-3 cancer xenograft growth without any toxicity in mice. Downregulation of cyclin D1 expression via a COX-2 dependent mechanism by Celecoxib could be a potential *in vivo* mechanism to inhibit ovarian cancer growth. Targeting cyclin D1 may be one of the molecular mechanisms restraining the growth of ovarian cancer by COX-2 inhibition. Celecoxib might inhibit the expression of cyclin D1 by a COX-2 dependent mechanism.

## Figures and Tables

**Table 1:** The Δ*CT* of VEGF in four groups. Molecular isoforms of VEGF are generated by alternative splicing, rendering proteins containing 189-, 165- and 121-amino acid residues. VEGF 189, 165 and 121 were routinely detected in this series of ovarian cancer.

	Control	Celecoxib (25 mg)	Celecoxib (100 mg)	5-FU
VEGF 121	3.55 ± 0.17	3.72 ± 0.16	4.91 ± 0.22	5.47 ± 0.19
VEGF 165	3.28 ± 0.21	5.00 ± 0.19	5.13 ± 0.19	6.12 ± 0.24
VEGF 189	4.72 ± 0.17	5.58 ± 0.12	5.70 ± 0.34	6.02 ± 0.29

## References

[1] Yokoyama Y Sakamoto T Sato S Saito Y Evaluation of cytoreductive surgery with pelvic and paraaortic lymphadenectomy and intermittent cisplatin-based combination chemotherapy for improvement of long-term survival in ovarian cancer Eur. J. Gynaecol. Oncol 1999 20 361 366 10609495

[2] Williams CS Mann M DuBois RN The role of cyclooxygenases in inflammation, cancer, and development Oncogene 1999 18 7908 7916 10630643 10.1038/sj.onc.1203286

[3] Dempke W Rie C Grothey A Schmoll HJ Cyclooxygenase-2: A novel target for cancer chemotherapy? J. Cancer Res. Clin. Oncol 2001 127 411 417 11469677 10.1007/s004320000225 PMC12164863

[4] Li S Miner K Fannin R Barrett JC Davis BJ Cyclooxygenase-1 and 2 in normal and malignant human ovarian epithelium Gynecol. Oncol 2004 92 622 627 14766256 10.1016/j.ygyno.2003.10.053

[5] Denkert C Köbel M Pest S Koch I Berger S Schwabe M Siegert A Reles A Klosterhalfen B Hauptmann S Expression of cyclooxygenase-2 is an independent prognostic factor in human ovarian carcinoma Am. J. Pathol 2002 160 893 903 11891188 10.1016/S0002-9440(10)64912-7 PMC1867167

[6] Erkinheimo TL Lassus H Finne P van Rees BP Leminen A Ylikorkala O Haglund C Butzow R Ristimäki A Elevated cyclooxygenase-2 expression is associated with altered expression of p53 and SMAD4, amplification of HER-2/neu, and poor outcome in serous ovarian carcinoma Clin. Cancer Res 2004 10 538 545 14760075 10.1158/1078-0432.ccr-0132-03

[7] Arico S Pattingre S Bauvy C Gane P Barbat A Codogno P Ogier-Denis E Celecoxib induces apoptosis by inhibiting 3-phosphoino-sitide-dependent protein kinase-1 activity in the human colon cancer HT-29 cell line J. Biol. Chem 2002 277 27613 27621 12000750 10.1074/jbc.M201119200

[8] Xin B Yokoyama Y Shigeto T Mizunuma H Anti-tumor effect of non-steroidal antiinflammatory drugs on human ovarian cancers Pathol. Oncol. Res 2007 13 365 369 18158574 10.1007/BF02940318

[9] Xin B Yokoyama Y Shigeto T Futagami M Mizunuma H Inhibitory effect of meloxicam, a selective cyclooxygenase-2 inhibitor, and Ciglitazone, a peroxisome proliferator-activated receptor gamma ligand, on the growth of human ovarian cancers Cancer 2007 110 791 800 17582802 10.1002/cncr.22854

[10] Uddin S Ahmed M Hussain A Assad L Al-Dayel F Bavi P Al-Kuraya KS Munkarah A Cyclooxygenase-2 inhibition inhibits PI3K/AKT kinase activity in epithelial ovarian cancer Int. J. Cancer 2010 126 386 394 10.1002/ijc.24757 19621391

[11] Ragel BT Jensen RL Gillespie DL Prescott SM Couldwell WT Celecoxib inhibits meningioma tumor growth in a mouse xenograft model Cancer 2007 190 588 597 17177201 10.1002/cncr.22441

[12] Basu GD Pathangey LB Tinder TL Lagioia M Gendler SJ Mukherjee P Cyclooxygenase-2 inhibitor induces apoptosis in breast cancer cells in an *in vivo* model of spontaneous metastatic breast cancer Mol. Cancer Res 2004 2 632 642 15561779

[13] Wang L Chen W Xie X He Y Bai X Celecoxib inhibits tumor growth and angiogenesis in an orthotopic implantation tumor model of human colon cancer Exp. Oncol 2008 30 42 51 18438340

[14] Leahy KM Ornberg RL Wang Y Zweifel BS Koki AT Masferrer JL Cyclooxygenase-2 inhibition by Celecoxib reduces proliferation and induces apoptosis in angiogenic endothelial cells *in vivo* Cancer Res 2002 62 625 631 11830509

[15] Wu GQ Xie D Yang GF Liao YJ Mai SJ Deng HX Sze J Guan XY Zeng YX Lin MC Kung HF Cell cycle-related kinase supports ovarian carcinoma cell proliferation via regulation of cyclin D1 and is a predictor of outcome in patients with ovarian carcinoma Int. J. Cancer 2009 125 2631 2642 19672860 10.1002/ijc.24630

[16] Barbieri F Lorenzi P Ragni N Schettini G Bruzzo C Pedullà F Alama A Overexpression of cyclin D1 is associated with poor survival in epithelial ovarian cancer Oncology 2004 66 310 315 15218299 10.1159/000078332

[17] Ferrara N Leung DW Cachianes G Winer J Henzel WL Purification and cloning of vascular endothelial growth factor secreted by pituitary folliculostellate cells Methods Enzymol 1991 198 391 405 1857232 10.1016/0076-6879(91)98040-d

[18] Denkert C Köbel M Pest S Koch I Berger S Schwabe M Siegert A Reles A Klosterhalfen B Hauptmann S Expression of cyclooxygenase 2 is an independent prognostic factor in human ovarian carcinoma Am. J. Pathol 2002 160 893 903 11891188 10.1016/S0002-9440(10)64912-7 PMC1867167

[19] Symowicz J Adley BP Woo MM Auersperg N Hudson LG Stack MS Cyclooxygenase-2 functions as a downstream mediator of lysophosphatidic acid to promote aggressive behavior in ovarian carcinoma cells Cancer Res 2005 65 2234 2242 15781636 10.1158/0008.5472.CAN-04-2781

[20] Worsley SD Ponder BA Davies BR Overexpression of cyclin D1 in epithelial ovarian cancers Gynecol. Oncol 1997 64 189 195 9038263 10.1006/gyno.1996.4569

[21] Masamha CP Benbrook DM Cyclin D1 degradation is sufficient to induce G1 cell cycle arrest despite constitutive expression of cyclin E2 in ovarian cancer cells Cancer Res 2009 69 6565 6572 19638577 10.1158/0008-5472.CAN-09-0913

[22] Vital-Reyes V Rodríguez-Burford C Chhieng DC Oelschlager DK Reyes-Fuentes A Barnes M Grizzle WE Celecoxib inhibits cellular growth, decreases Ki-67 expression and modifies apoptosis in ovarian cancer cell lines Arch. Med. Res 2006 37 689 695 16824926 10.1016/j.arcmed.2005.11.014

[23] Arnold A Papanikolaou A Cyclin D1 in breast cancer pathogenesis J. Clin. Oncol 2005 23 4215 4224 15961768 10.1200/JCO.2005.05.064

[24] Schiffmann S Maier TJ Wobst I Janssen A Corban-Wilhelm H Angioni C Geisslinger G Grösch S The anti-proliferative potency of celecoxib is not a class effect of coxibs Biochem. Pharmacol 2008 76 179 187 18547544 10.1016/j.bcp.2008.04.017

[25] Soh JW Kazi JU Li H Thompson WJ Weinstein IB Celecoxib-induced growth inhibition in SW480 colon cancer cells is associated with activation of protein kinase G Mol. Carcinog 2008 47 519 525 18163459 10.1002/mc.20409

[26] D’Andrilli G Kumar C Scambia G Giordano A Cell cycle genes in ovarian cancer: steps toward earlier diagnosis and novel therapies Clin. Cancer Res 2004 10 8132 8141 15623586 10.1158/1078-0432.CCR-04-0886

[27] Macaluso M Paggi MG Giordano A Genetic and epigenetic alterations as hallmarks of the intricate road to cancer Oncogene 2003 22 6472 6478 14528270 10.1038/sj.onc.1206955

[28] Narayanan BA Condon MS Bosland MC Narayanan NK Reddy BS Suppression of *N*-methyl-*N*-nitrosourea/testosterone-induced rat prostate cancer growth by celecoxib: effects on cyclooxygenase-2, cell cycle regulation, and apoptosis mechanism(s) Clin. Cancer Res 2003 15 3503 3513 12960143

[29] Gastman BR Apoptosis and its clinical impact Head Neck 2001 23 409 425 11295816 10.1002/hed.1052

[30] Song YC Kim SH Juhnn YS Song YS Apoptotic effect of celecoxib dependent upon p53 status in human ovarian cancer cells Ann. N.Y. Acad. Sci 2007 1095 26 34 17404014 10.1196/annals.1397.004

[31] Olson TA Mohanraj D Carson LF Ramakrishnan S Vascular permeability factor gene expression in normal and neoplastic ovaries Cancer Res 1994 54 276 280 8261452

[32] Yoneda J Kuniyasu H Crispens MA Price JE Bucana CD Fidler IJ Expression of angiogenesis-related genes and progression of human ovarian carcinomas in nude mice J. Natl. Cancer Inst 1998 90 447 454 9521169 10.1093/jnci/90.6.447

[33] Hartenbach EM Olson TA Goswitz JJ Mohanraj D Twiggs LB Carson LF Ramakrishnan S Vascular endothelial growth factor expression and survival in human epithelial ovarian carcinomas Cancer Lett 1997 121 169 175 9570355 10.1016/s0304-3835(97)00350-9

[34] Paley PJ Staskus KA Gebhard K Mohanraj D Twiggs LB Carson LF Ramakrishnan S Vascular endothelial growth factor expression in early stage ovarian carcinomas Cancer 1997 80 98 106 9210714 10.1002/(sici)1097-0142(19970701)80:1<98::aid-cncr13>3.0.co;2-a

[35] Yamamoto S Konishi I Mandai M Kuroda H Komatsu T Nanbu K Sakahara H Mori T Expression of vascular endothelial growth factor (VEGF) in epithelia ovarian neoplasms: correlation with clinicopathology and patient survival and analysis of serum VEGF levels Br. J. Cancer 1997 76 1221 1227 9365173 10.1038/bjc.1997.537 PMC2228134

[36] Lee JS Choi YD Lee JH Nam JH Choi C Lee MC Park CS Juhng SW Min KW Expression of cyclooxygenase-2 in epithelial ovarian tumors and its relation to vascular endothelial growth factor and p53 expression Int. J. Gynecol. Cancer 2006 16 247 253 16515599 10.1111/j.1525-1438.2006.00477.x

[37] Fujimoto J Toyoki H Sakaguchi H Jahan I Alam SM Tamaya T Clinical implications of expression of cyclooxygenase-2 related to angiogenesis in ovarian cancer Oncol. Rep 2006 15 21 25 16328030

[38] Gavrieli Y Sherman Y Ben-Sasson SA Identification of programmed cell death *in situ* via specific labeling of nuclear DNA fragmentation J. Cell Biol 1992 119 493 501 1400587 10.1083/jcb.119.3.493 PMC2289665

[39] Del-Vecchio MT Leoncinel L Buerdi K Kraft R Megha T Barbini P Tosi P Cottier H Diffuse controcytic and/or centroblastic malignant non-Hodgkins lymphomas: comparison of mitotic and pyknotic (apoptotic) indices Int. J. Cancer 1991 47 38 43 1985876 10.1002/ijc.2910470108

